# Genetics and epigenetics in tumor necrosis factor-alpha (*TNF-α*) and transmembrane 6 superfamily member 2 (*TM6SF2*) in alcohol induced liver cirrhosis

**DOI:** 10.3389/fmed.2026.1866012

**Published:** 2026-07-29

**Authors:** Bhagyalakshmi Shankarappa, Jayant Mahadevan, Pratima Murthy, Biju Viswanath, Harshad Devarbhavi, Sanjeev Jain, Meera Purushottam

**Affiliations:** 1 Molecular Genetics Lab, National Institute of Mental Health and Neurosciences (NIMHANS), Bangalore, India; 2 Department of Psychiatry, National Institute of Mental Health and Neurosciences (NIMHANS), Bangalore, India; 3 Department of Gastroenterology, St. John’s Medical College Hospital, Bangalore, India

**Keywords:** alcohol dependence, alcohol liver cirrhosis, DNA methylation, TM6SF2, TNF-alpha

## Abstract

**Background:**

Alcohol dependence and cirrhosis are key outcomes of increased alcohol use, with genetic factors increasingly implicated. A functional promoter variant (rs361525) in the *TNF-α* gene may contribute to ALD pathogenesis, while a loss-of-function variant (rs58542926) in *TM6SF2* modifies liver disease progression. We aimed to study these SNPs and assess DNA methylation at *TM6SF2* loci in individuals with and without alcohol-related cirrhosis.

**Methods:**

The study included men (*N* = 243) with alcohol dependence with cirrhosis (AUD-C+ve) and without cirrhosis (AUD-C-ve), based on ICD-10 criteria, recruited from SJMCH. Fibroscan and/or sonography (LSM < 14 kPa) ruled out severe fibrosis. Genotyping was performed for TNFα (rs361525) and *TM6SF2* (rs58542926). Genomic DNA (*N* = 100) underwent bisulfite conversion followed by pyrosequencing at *TM6SF2* loci. Methylation levels were calculated separately for individual CpG sites and as the mean of four CpG sites. Group differences were assessed using unpaired *t*-tests, and genetic models were applied based on risk allele status.

**Results:**

Genotype and allele frequencies at both loci were comparable between AUD-C+ve and AUD-C–ve groups. *TM6SF2* methylation was significantly reduced in the AUD-C+ve group (uncorrected *p* = 0.03). A trend was also noted for TNF-genotype with A-carriers having lower *TM6SF2* global methylation levels (*p* = 0.06). We did not observe any differences in genotype and allele frequencies for both *TM6SF2* and *TNFα* variants. Duration of alcohol consumption was significantly associated with *TM6SF2* methylation (*p* = 0.02).

**Conclusion:**

*TM6SF2* hypomethylation in AUD-C+ve individuals may influence lipid metabolism and contribute to liver injury and HCC progression. Reduced methylation among *TNF-α* risk allele carriers suggests a potential gene–epigenetic interaction. Overall, higher alcohol exposure and genetic susceptibility may together predispose to worsening alcohol-related cirrhosis.

## 1 Introduction

Alcohol-associated liver disease (ALD) encompasses a spectrum of liver injuries, ranging from simple steatosis to steatohepatitis, fibrosis, cirrhosis, and hepatocellular carcinoma (HCC) (1, 2). While chronic and excessive alcohol consumption is the primary driver, a subset of individuals rapidly progresses to advanced liver disease, highlighting the critical role of host genetic and epigenetic factors in determining individual susceptibility (3, 4).

Tumor Necrosis Factor-alpha (*TNF-α*) is a master pro-inflammatory cytokine central to the pathogenesis of ALD. Ethanol metabolism disrupts gut barrier integrity, leading to endotoxemia, which in turn activates hepatic Kupffer cells to produce excessive *TNF-α* (5, 6). This sustained inflammatory response promotes hepatocyte apoptosis, necroinflammation, and contributes to the progression of liver injury (7). A functional promoter variant in the *TNF-α* gene (rs361525) has been extensively studied for its association with altered inflammatory responses and susceptibility to various inflammatory and liver diseases (8).

More recently, a loss-of-function variant in the *Transmembrane 6 Superfamily Member 2* (*TM6SF2*) genes (rs58542926, E167K) has been identified as a key genetic modifier of liver disease progression, both in metabolic dysfunction-associated steatotic liver disease (MASLD) and ALD (9). The TM6SF2 protein is predominantly expressed in the liver and intestine and plays a crucial role in the secretion and metabolism of very-low-density lipoproteins (VLDL) (10, 11). The E167K variant impairs this function, leading to intrahepatic retention of triglycerides and cholesterol esters, thereby promoting steatosis and creating a lipotoxic environment that drives further liver injury (12, 13).

Epigenetic modifications, particularly DNA methylation, serve as a mechanistic interface between genetic predisposition and environmental exposures like alcohol, potentially explaining the variable phenotypic expression of disease (14, 15). Alcohol consumption is known to alter global and gene-specific methylation patterns by interfering with one-carbon metabolism and methyl donor availability, thereby influencing gene expression and disease phenotype (16, 17). However, the relationship between *TM6SF2* promoter methylation, its genetic variants, and the inflammatory milieu in ALD remains largely unexplored.

This study aimed to: (1) Analyze the association of *TNF-α* (rs361525) and *TM6SF2* (rs58542926) SNPs with alcohol-associated cirrhosis; (2) Investigate DNA methylation levels at the *TM6SF2* locus in patients with and without cirrhosis; and (3) Elucidate the interplay between genotype, alcohol consumption, and *TM6SF2* methylation.

## 2 Materials and methods

### 2.1 Study population

The study included 243 male participants recruited from the Gastroenterology and Psychiatry services at St. John’s Medical College Hospital from June 2017 to March 2020, participants were divided into two groups:

AUD-C+ve (*n* = 136): Patients with Alcohol Dependence (based on ICD-10 criteria) and confirmed cirrhosis (clinical, biochemical, and radiological/sonographic evidence).

AUD-C-ve (*n* = 107): Patients with Alcohol Dependence but without cirrhosis or severe fibrosis. The absence of significant fibrosis was confirmed by Fibroscan (Liver Stiffness Measurement, LSM < 14 kPa) and/or sonographic findings.

Severity of alcohol-related liver cirrhosis was assessed using the Child–Pugh score, which incorporates serum bilirubin, serum albumin, prothrombin time/INR, ascites, and hepatic encephalopathy. Total scores (5–15) classified patients as Child–Pugh A (5, 6), B (7–9), or C (10–15), indicating compensated to severely decompensated cirrhosis. Additionally, the Model for End-Stage Liver Disease (MELD) score, calculated from serum bilirubin, INR, and serum creatinine, was used to assess disease severity, with higher scores (range 6–40) indicating greater severity.

The study was approved by the institutional ethics committee, and informed consent was obtained from all participants.

### 2.2 Genotyping

Genomic DNA was extracted from peripheral blood samples. Genotyping for *TNF-α* (rs361525) and *TM6SF2* (rs58542926) SNPs were performed using Polymerase Chain Reaction-Restriction Fragment Length Polymorphism (PCR-RFLP) with appropriate restriction enzymes (*Msp*I for rs361525 and *Hyp*I for rs58542926). Genotypes were confirmed by sequencing a random sample.

### 2.3 DNA methylation analysis

A subset of 100 genomic DNA samples (50 from each group) underwent bisulfite conversion using the EZ DNA Methylation-Lightning™ Kit (Zymo Research). The *TM6SF2* locus was amplified, and quantitative DNA methylation analysis at four contiguous CpG sites was performed using pyrosequencing on a PyroMark Q48 system (Qiagen). The average methylation value across the four CpG sites was used for the primary statistical analyses, while individual CpG sites were also analyzed separately to investigate site-specific methylation patterns and associations. Inter-assay reproducibility was assessed by repeat pyrosequencing of a randomly selected subset comprising 10% of samples. Bisulfite conversion efficiency was monitored using internal controls, and only samples demonstrating a conversion efficiency of >95% were included for downstream analyses.

**Figure 1:**
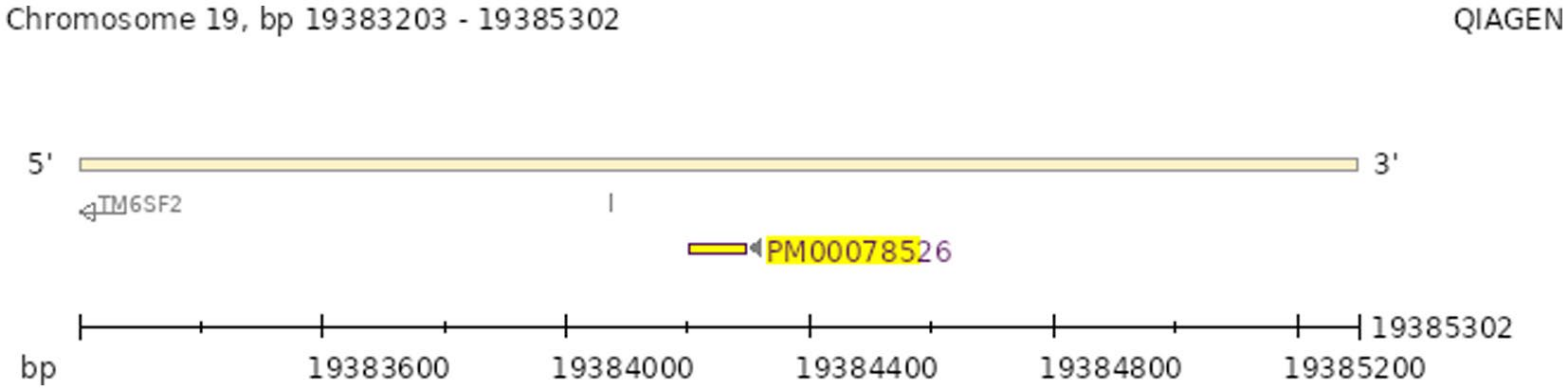
*TM6SF2* methylation (Gene Globe ID - PM00078526) – region of interest. Chromosome 19, bp 19,383,203–19,385,302. Strand of interest (sense). G**CG**GGA**CG**GCCATTGGAGA**CG**AGGGTGT**CG**A. Strand of interest after bisulphite conversion. G**CG**GGA**CG**GTTATTGGAGA**CG**AGGGTGT**CG**A.

The genomic location of the four CpG sites was annotated using the UCSC Genome Browser and Ensembl, confirming that all four sites lie within a CpG island overlapping the promoter/5′ regulatory region of *TM6SF2* (Figure 1).

### 2.4 Statistical analysis

Hardy–Weinberg equilibrium (HWE) was assessed for both SNPs in the AUD-C-ve reference group using the chi-square goodness-of-fit test. The differences in genotype and allele frequencies between groups were evaluated using chi-square tests.

To evaluate the distribution of methylation values, Shapiro–Wilk tests were performed to assess normality prior to subsequent statistical analyses. The Mann–Whitney U test was used to compare median DNA methylation levels between groups, followed by correction for age, quantity of alcohol consumed per day and years of drinking. Benjamini–Hochberg false discovery rate (FDR) correction was applied across all methylation comparisons.

Linear regression models were then fitted to test the association between TM6SF2 methylation levels (dependent variable) and *TNF-α* genotype, correcting for age, quantity of alcohol consumed per day and years of drinking in the total sample and AUD-C+ve and AUD-C-ve groups, separately.

Logistic regression models were fitted to test the association between diagnosis (dependent variable) and the interaction between *TM6SF2* methylation and *TM6SF2* genotype and TNF-*α* genotypes. All Analyses were performed using R v4.0.5.

## 3 Results

The clinical and demographic characteristics of the individuals selected in the study are tabulated in Table 1. We observed that alcohol dependence with cirrhosis group (AUD-C+ve) had used lesser quantities of alcohol (mean ± SD = 13.7 ± 6.8 units/day) compared to the alcohol dependence without cirrhosis group (AUD-C-ve; mean ± SD = 16.1 ± 7.7 units/day; *p* = 0.01). AUD-C+ve group had also been consuming alcohol for a shorter duration comparatively (16 ± 7 years vs. 18 ± 8 years) and had a later age of onset of drinking (29 ± 8 years vs. 23 ± 7 years; *p* < 0.001) compared to AUD-C-ve group (Table 1). The MELD score values for the AUD-C+ve group were 23.4 ± 7.5 and ranged from 9 to 40. Child-Pugh scores indicated a severity ranging from moderately severe to most severe liver disease (mean ± SD of 11.1 ± 1.7 Child B/C).

**Table 1:** Demographic data.

Variables	Whole sample set (*N* = 243)		
	AUDC-ve (*N* = 107)	AUDC+ve (*N* = 136)	*p*-value
	Mean ± SD (range)	Mean ± SD (range)	
Age	41 ± 10	45 ± 9	0.01*
Years of drinking	18 ± 8 (8–36)	16 ± 7 (1–35)	0.11
Alcohol intake in units	16.1 ± 7.7 (3–42)	13.7 ± 6.8 (2–36)	0.01*
Age at onset of drinking	23 ± 7 (18–46)	29 ± 8 (6–51)	<0.001*
Total bilirubin	1.32 ± 1.06 (0.27–5.22)	8.44 ± 8.90 (0.40–41.93)	<0.001*
Direct bilirubin	0.40 ± 0.30 (0–2)	5.83 ± 6.67 (0–29)	<0.001*
AST	94.9 ± 84.4 (11–436)	112.4 ± 102.5 (17–907)	0.15
ALT	66.5 ± 46.4 (10–206)	50.32 ± 46.9 (8–357)	0.009*
AST/ALT ratio	1.5 ± 1.12 (0.33–7.03)	2.58 ± 1.29 (0.28–7.71)	<0.001*
GGT	273.1 ± 270 (13–1,291)	298.3 ± 411 (12–3,006)	0.58
ALP	106.0 ± 56.5 (28–482)	159 ± 105 (31–1,018)	<0.001*
GGT/ALP ratio	2.64 ± 2.41 (0.16–10.94)	2.10 ± 3.34 (0.08–22.7)	0.15
Total protein	7.14 ± 0.73 (5–10)	6.64 ± 0.99 (4–9)	<0.001*
Albumin	3.9 ± 0.74 (2–9)	2.52 ± 0.80 (1–5)	<0.001*
Hemoglobin	14.4 ± 1.9 (10–18)	10.56 ± 2.65 (4–17)	<0.001*
MCV	93.30 ± 6.9 (80–110)	94.40 ± 9.9 (61–115)	0.54
Homocysteine	15.6 ± 12.5 (4.6–96.9)	15 ± 8.7 (3–40.5)	0.72

Comparison of sociodemographic, clinical variables and biochemical parameters between individuals with AUD with and without cirrhosis in the entire sample. * Indicates significance *p* < 0.05.

AUD, alcohol dependence; AUD-C+ve, alcohol dependence with cirrhosis; AUD-C−ve, alcohol dependence without cirrhosis; ALD, alcohol-related liver disease; TNF-α, tumour necrosis factor-alpha; TM6SF2, transmembrane 6 superfamily member 2; SNP, single nucleotide polymorphism; HWE, Hardy–Weinberg equilibrium; OR, odds ratio; CI, confidence interval; MELD, model for end-stage liver disease; LSM, liver stiffness measurement; AST, aspartate aminotransferase; ALT, alanine aminotransferase; GGT, gamma-glutamyl transferase; ALP, alkaline phosphatase; MCV, mean corpuscular volume; SD, standard deviation; FDR, false discovery rate; CpG, cytosine-phosphate-guanine; PCR-RFLP, polymerase chain reaction–restriction fragment length polymorphism; HCC, hepatocellular carcinoma; VLDL, very-low-density lipoprotein. Bold values indicate statistically significant differences (*p* < 0.05).

### 3.1 Genetic association analysis

The distribution of genotypes and alleles for both *TNF-α* (rs361525) and *TM6SF2* (rs58542926) was similar between the AUD-C+ve and AUD-C-ve groups, showing no significant association with cirrhosis status in this cohort.

### 3.2 *TM6SF2* DNA methylation analysis

**Figure 2:**
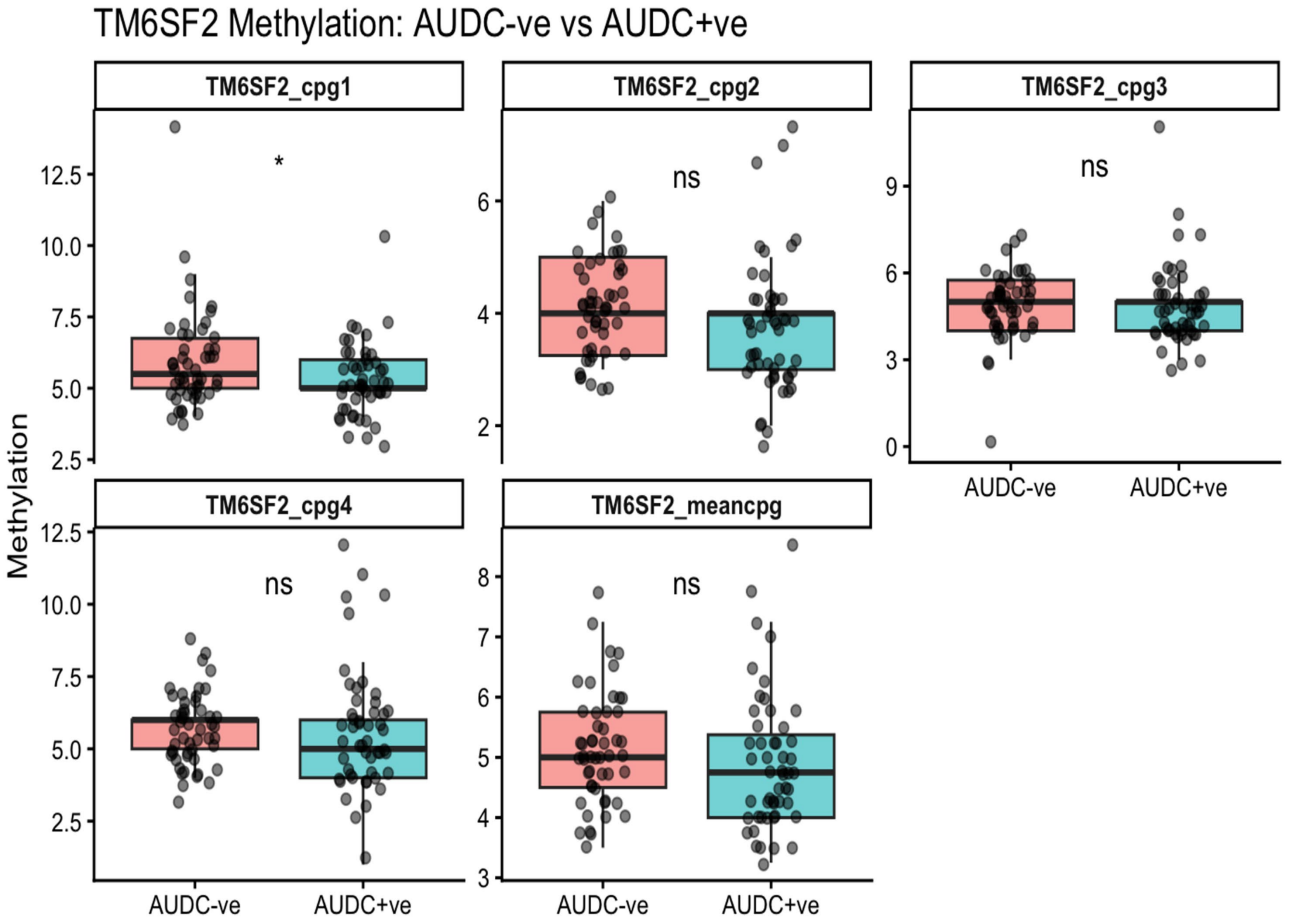
*TM6SF2* DNA Methylation. **TM***6SF2* DNA methylation levels in AUD-C + ve and AUD-C-ve individuals. The horizontal line represents the median value. * Indicates significance *p* < 0.05.

The AUD-C+ve group demonstrated significantly lower methylation levels at the CpG1 site of the TM6SF2 locus compared to the AUD-C-ve group (*p* = 0.03; Figure 2); however, significance did not survive FDR correction (Supplementary Tables 1, 2).

A significant positive association was noted between years of drinking and *TM6SF2* CpG 1 methylation levels (estimate = 0.0563, 95% CI [0.0088, 0.1038], *p* = 0.02) in the full sample. A trend was also noted for TNF- genotype with A-carriers having lower *TM6SF2* global methylation levels (estimate = −0.4434, 95% CI [−0.9143, 0.0275], *p* = 0.06; Supplementary Figure 1). A significant association was also noted for the TNF-ɑ genotype with A-carriers having lower *TM6SF2* global methylation levels (estimate = −0.6075, 95% CI [−1.2149, 0.0000], *p* = 0.05) and *TM6SF2* CpG4 methylation levels (estimate = −0.8914, 95% CI [−1.7151, −0.0677], *p* = 0.03) in the AUDC-ve group (Supplementary Figure 3).

There was no association of diagnosis to the interaction effects of *TM6SF2* or TNF-ɑ genotype and *TM6SF2* methylation (Supplementary Tables 3, 4).

## 4 Discussion

This study provides novel evidence for an epigenetic mechanism involving *TM6SF2* hypomethylation in the progression of ALD. While the genetic variants themselves were not directly associated with cirrhosis status in our cohort, their functional impact appears to be mediated through alterations in the epigenetic landscape, which are exacerbated by alcohol consumption.

Our key finding is hypomethylation at the *TM6SF2* locus in patients with established cirrhosis (AUD-C+ve) compared to those without (AUD-C-ve), although this association did not survive correction for multiple testing (Table 2). Nevertheless, the direction of this effect is biologically plausible, as DNA hypomethylation in gene promoter or regulatory regions is often associated with enhanced gene expression (18). While mRNA expression levels were not measured, hypomethylation at regulatory regions may enhance *TM6SF2* transcription (18, 19). However, this potential increase in gene expression may not translate into functional activity, as the E167K variant produces a loss-of-function protein that impairs lipid export and promotes hepatic lipid accumulation (11, 20).

**Table 2:** Genotype and allele frequency for *TM6SF2 and TNFα* SNPs.

Genotype/allele frequency	AUD-C+ve *N* (%)	AUD-C-ve *N* (%)	OR (95%CI) *p*-value
*TM6SF2* (rs58542926)			
Genotype frequency	*N* = 124	*N* = 103	
CC	99 (80)	82 (80)	*p* = 0.71
CT	15 (12)	15 (14)	
TT	10 (8)	6 (6)	
Allele frequency	2n = 248	2n = 206	
C	213 (86)	179 (87)	1.09 (0.63–1.87) *p* = 0.75
T	35 (14)	27 (13)	
*TNFα* (rs361525)			
Genotype frequency	*N* = 131	*N* = 105	
GG	107 (82)	82 (78)	*p* = 0.2
GA	20 (15)	15 (14)	
AA	4 (3)	8 (8)	
Allele frequency	2n = 262	2n = 210	
G	234 (89)	179 (85)	0.69 (0.40–1.19) *p* = 0.18
A	28 (11)	31 (15)	

Therefore, increased expression of dysfunctional protein could amplify its deleterious effects on VLDL secretion, leading to greater hepatic lipid accumulation and lipotoxicity, thereby accelerating liver injury (21).

This hypothesis is strengthened by our findings within the AUD-C-ve group: carriers of the A (risk) allele exhibited significantly lower *TM6SF2* global methylation (estimate = −0.6075, 95% CI [−1.2149, 0.0000], *p* = 0.05) and CpG4 methylation (estimate = −0.8914, 95% CI [−1.7151, −0.0677], *p* = 0.03) compared to non-carriers. This suggests a pre-existing epigenetic predisposition in genetically vulnerable individuals. We acknowledge the apparent biological inconsistency that this *TNF-α* genotype-methylation association was observed only in the AUD-C-ve group and not in the AUD-C+ve group. One possible explanation is that this represents a pre-cirrhotic epigenetic signature that is subsequently masked or overridden by the more global epigenetic disruption associated with established cirrhosis, such that genotype-specific effects on methylation become less detectable once advanced disease-related hypomethylation predominates (22). This interpretation remains speculative and would require prospective longitudinal validation, ideally with serial methylation measurements across the transition from non-cirrhotic to cirrhotic disease states within the same individuals.

Furthermore, association was observed between *TM6SF2* methylation levels and duration of drinking within the ADS group, suggesting an exposure-dependent effect on *TM6SF2* methylation. Consistent with this, *TM6SF2* expression has been reported to be reduced in steatotic liver and to vary with disease progression, indicating dynamic regulation during liver injury (11, 20, 23). Alcohol is known to interfere with one-carbon metabolism, depleting S-adenosylmethionine (SAM), the universal methyl donor, leading to global hypomethylation (11, 24).

The role of *TNF-α*, while not directly linked to methylation changes in our analysis, remains central to the pathological process. As concluded, ethanol-induced *TNF-α* promotes inflammation and insulin resistance, enhancing SREBP-1c mediated *de novo* lipogenesis. This creates a lipid-rich environment where the *TM6SF2* dysfunction has its maximum damaging effect.

The interplay likely creates a vicious cycle: inflammation and lipotoxicity promote cellular stress and changes in methylation, which further disrupt lipid metabolism and fuel more inflammation, driving progression toward cirrhosis and hepatocellular carcinoma (6).

The lack of association between *TM6SF2* rs58542926 and cirrhosis in our cohort, despite strong associations reported elsewhere, may reflect ethnic-specific allele frequency and linkage disequilibrium differences between this South Indian population and the predominantly European cohorts in prior studies (25), as well as our modest sample size and potential selection bias from tertiary-referral recruitment enriching both arms for severe disease. Additionally, given the polygenic architecture of ALD, individual SNP effects are likely modest and context-dependent rather than independently decisive (26).

### 4.1 Study limitations

The sample size, particularly for the methylation subset, is modest. The cross-sectional design allows for the identification of associations but not causal inferences. Functional validation, such as measuring TM6SF2 mRNA and protein expression in relation to methylation status, was not performed and is a critical next step.

A key limitation of this study is its exclusive focus on male participants, which limits the generalizability of these findings to women. Given known sex-based differences in alcohol metabolism, hormonal regulation, and epigenetic patterning, future studies in female and mixed-sex cohorts are needed to establish whether the genetic and epigenetic associations observed here are sex-independent. Future studies employing genome-wide approaches in larger cohorts may provide a more comprehensive view of the underlying molecular landscape.

## 5 Conclusion

In conclusion, our data moves beyond simple association and highlights a dynamic gene–environment interaction in ALD. We propose a model whereby heavy alcohol consumption induces hypomethylation at the *TM6SF2* locus. In individuals carrying the E167K loss-of-function variant, this epigenetic dysregulation may promote the expression of a dysfunctional protein, severely compromising VLDL secretion and cholesterol metabolism. This, superimposed on an alcohol-induced pro-inflammatory and pro-steatotic background driven by factors like *TNF-α*, significantly exacerbates the risk of progressive liver damage and cirrhosis. *TM6SF2* DNA methylation could serve as a potential biomarker for identifying AUD patients at highest risk for progression, allowing for targeted interventions.

## Data Availability

The original contributions presented in the study are included in the article/Supplementary material. The datasets generated and analysed during this study are available from the corresponding author upon reasonable request.
