# Supplementary material for: Genetics and epigenetics in tumor necrosis factor-alpha (TNF-α) and transmembrane 6 superfamily member 2 (TM6SF2) in alcohol induced liver cirrhosis
Source: Front Med (Lausanne). 2026 Jul 29;13:1866012. doi: 10.3389/fmed.2026.1866012 (PMC13461408; doi:10.3389/fmed.2026.1866012)
Supplement: Supplementary file 1 [file Table_1.DOCX]

Supplementary Information

Supplementary Table 1: Differences in TM6SF2 methylation between patients in AUDC+ve and AUDC-ve groups

| Variable | AUDC+ve | AUDC-ve | Median Difference | p-value (uncorr) | p-value (FDR) |
| --- | --- | --- | --- | --- | --- |
| TM6SF2 Methylation | 4.75 (1.375) | 5.0 (1.25) | -0.25 | 0.0922 | 0.154 |
| TM6SF2 CpG1 Methylation | 5.0 (1.00) | 5.5 (1.75) | -0.50 | 0.0748 | 0.154 |
| TM6SF2 CpG2 Methylation | 4.0 (1.00) | 4.0 (1.75) | 0.00 | 0.0319 | 0.154 |
| TM6SF2 CpG3 Methylation | 5.0 (1.00) | 5.0 (1.75) | 0.00 | 0.3040 | 0.379 |
| TM6SF2 CpG4 Methylation | 5.0 (2.00) | 6.0 (1.00) | -1.00 | 0.5260 | 0.526 |

Supplementary Table 2: Differences in TM6SF2 methylation between patients in AUDC+ve and AUDC-ve groups after adjusting for age, quantity and years of drinking

| Variable | AUDC+ve | AUDC-ve | Median Difference | p-value (uncorr) | p-value (FDR) |
| --- | --- | --- | --- | --- | --- |
| TM6SF2 Methylation | 4.75 (1.375) | 5.0 (1.25) | -0.25 | 0.6231 | 0.779 |
| TM6SF2 CpG1 Methylation | 5.0 (1.00) | 5.5 (1.75) | -0.50 | 0.2254 | 0.564 |
| TM6SF2 CpG2 Methylation | 4.0 (1.00) | 4.0 (1.75) | 0.00 | 0.2008 | 0.564 |
| TM6SF2 CpG3 Methylation | 5.0 (1.00) | 5.0 (1.75) | 0.00 | 0.9145 | 0.914 |
| TM6SF2 CpG4 Methylation | 5.0 (2.00) | 6.0 (1.00) | -1.00 | 0.5225 | 0.779 |

Supplementary Table 3: Interaction between TNF-α Genotype and TM6SF2 methylation in patients in AUDC+ve and AUDC-ve groups

| Predictor Variable | β | SE | Z value | OR (95% CI) | P value |
| --- | --- | --- | --- | --- | --- |
| Intercept | 1.7290 | 1.1953 | 1.446 | 5.635 (0.576, 65.959) | 0.148 |
| TNF-α Genotype | -2.1027 | 2.4988 | -0.841 | 0.122 (0.001, 17.222) | 0.400 |
| TM6SF2 Methylation | -0.3314 | 0.2287 | -1.449 | 0.718 (0.447, 1.109) | 0.147 |
| TNF-α Genotype* TM6SF2 Methylation | 0.4116 | 0.5165 | 0.797 | 1.509 (0.540, 4.355) | 0.425 |

Supplementary Table 4: Interaction between TM6SF2 Genotype and TM6SF2 methylation in patients in AUDC+ve and AUDC-ve groups

| Predictor Variable | β | SE | Z value | OR (95% CI) | P value |
| --- | --- | --- | --- | --- | --- |
| Intercept | 1.8249 | 1.2954 | 1.409 | 6.202 (0.520, 87.948) | 0.159 |
| TM6SF2 Genotype | -1.3807 | 2.3085 | -0.598 | 0.251 (0.002, 26.824) | 0.550 |
| TM6SF2 Methylation | -0.3830 | 0.2567 | -1.492 | 0.682 (0.401, 1.111) | 0.136 |
| TM6SF2 Genotype* TM6SF2 Methylation | 0.3110 | 0.4496 | 0.692 | 1.365 (0.548, 3.371) | 0.489 |

Supplementary Figure 1: Multivariable predictors of TM6SF2 methylation in the full sample

Legend: A significant positive association was noted between years of drinking and TM6SF2 CpG 1 methylation levels (Estimate = 0.0563, 95% CI (0.0088, 0.1038), p = 0.02) in the full sample. A trend was also noted for TNF-α genotype with A-carriers having lower TM6SF2 global methylation levels (Estimate = -0.4434, 95% CI (-0.9143, 0.0275), p = 0.06)

Supplementary Figure 2: Multivariable predictors of TM6SF2 methylation in the AUDC+ve sample

Supplementary Figure 3: Multivariable predictors of TM6SF2 methylation in the AUD-C-ve sample

Legend: A significant association was noted for TNF-α genotype with A-carriers having lower TM6SF2 global methylation levels (Estimate = -0.6075, 95% CI (-1.2149, 0.0000), p = 0.05) and TM6SF2 CpG4 methylation levels (Estimate = -0.8914, 95% CI (-1.7151, -0.0677), p = 0.03)
